# Some Thoughts on Crown and Bridge-Work

**Published:** 1890-09

**Authors:** W. Mitchell

**Affiliations:** London, England.


AMERICAN JOURNAL

—OF—

DENTAL SCIENCE.

VOL. XXIV. BALTIMORE, SEPTEMBER, 1890. No. 5.

ARTICLE I.

### SOME THOUGHTS ON CROWN AND BRIDGE-WORK.

BY W. MITCHELL, D. D. S., LONDON, ENGLAND.

**Read at the Meeting of the American Dental Society of Europe, August, 1889.**

*Mr. President and Gentlemen:*—It is not so much the purpose of this paper to present anything new as it is to sift out and secure to us that which will prove of most value in every-day practice, and be a guide to those commencing in this branch of operative dentistry, whereby they may trace an outline of procedure that will insure a minimum of unsatisfactory experiences, which are nearly always associated with our earliest efforts, and secure a maximum of satisfactory operations, both to the patient and to the operator.

Crown-work, in the abstract, has passed through possibly more stages of evolution than any other of our methods of restoring facial contour, or defective articulating or masticating power; and the outcome of the vast amount of thought and experiment that has been expended upon it is manifest in the various and numerous systems at our selection. The result is for good, for it is hardly possible to conceive of a case where the tooth or root is solid, and the adjacent tissues in a warrantable condition, where some kind of crown may not be used with comfort and benefit to the patient. Roots may be rendered more healthy and more in harmony with adjacent structures, and need considering much less as a factor in peripheral irritation, when properly treated and crowned and restored to legitimate use than when left excised and in a ragged condition, as a supposed support to a plate that frequently is a match for them in its filthy and unwholesome condition, a state of affairs brought about by wilful culpability by the addition of a necessarily ill-adapted artificial appliance to an already unsanitary and vitiated mouth.

To bridge-work, as frequently made, the foregoing remarks apply with particular emphasis; and while bridge-work in selected cases is undoubtedly a boon to the patient and a credit to the dentist, its common or indiscriminate use will undermine its real advantages and cause it to be looked upon with distrust and loathing, the first proposition promoted by lack of judgment and discretion, and the second by an ignoring or inability to recognize the fine mechanical manipulative requirements necessary to its perfect production and adjustment. Here I might mention, I deprecate most earnestly the indiscriminate excision of sound teeth, and drilling for anchorages to secure supposed supports for bars and footings; for in the one case very likely a perfectly sound tooth has been sacrificed, and in the other all the worst elements of an approximal cavity have been promoted, without any of the subsequent advantages of isolation, as is the case with a well polished and self-cleansing surface of an ordinary filling. Already, in England and America, the charlatans who pose upon their capacity for "crown, bar, and bridge-work" are constructing animated sewers whose vitalities are being sapped by the indiscriminate insertion of these—in many cases—"fearfully and wonderfully-made" appliances.

In my opinion, we have in bridge-work a valuable adjunct to our prosthetic *repertoire*, and one which, in capable and judicious hands, may be productive of much good; that the reverse of this is also true, it is scarcely necessary for me to suggest to my present audience.

I wish to lay special stress upon the necessity of carefully diagnosticating a case where a piece of bridge or bar-work is to be inserted, for, unless the diagnosis is a correct one, it necessarily follows the prognosis will be proportionately unfavorable. This applies more directly to bridge-work than to the adjustment of crowns, although with either, the requirements of the case as regards position, work to be done, adjacent and antagonizing teeth, all these points should be carefully noted, when a selection from the various methods should be made that is considered best suited to the case.

After some considerable experience in this class of operations, I have found the half cap, as still used by many, to be worse than no cap at all, affording, as it does, an exit for the cement or other retaining mediums, and subsequently a receptacle for food and secretions that rapidly decompose, making, what should be a thing of beauty and service, a veritable whited or gilded sepulchre.

For molars and second bicuspids the all-gold crown stands pre-eminently at the head of the list, possessing at once the greatest possible range in adaptability to more or less broken-down roots, restoration of masticating surface, cleanliness, and durability. It will thus be seen, if my claims are well founded, the gold-cap crown leaves little to be desired.

From an æsthetic stand-point it is inartistic where exposed to view; but if I had to choose between some of the miserable abortions called artificial dentures, superimposed upon roots reeking with morbid products, as we frequently see them, and alleged to be artistic, I should unhesitatingly prefer the inartistic and cleanly to the artistic and filthy.

But right here we are afforded an opportunity of combining utility with art, and securing a perfect result, for, by utilizing the principle of the gold-cap crown,— *i. e.*, a cap with a porcelain crown or facing,—we have a combination that leaves little to be desired. The Logan crown has been utilized this way, and I believe with very satisfactory results. I believe a solid porcelain tooth makes a much more desirable crown than the plate or flat tooth, for they can be adjusted to the cap with the minimum of heat, and are much less liable to come to grief in ordinary use than are the flat or plate teeth; this being the case, about five years ago I devised a crown and attachment, using Ash's tube teeth, that admitted of a range of adaptability that has proved very satisfactory, their colors leaving little to be desired; and while only having used a limited number of these crowns, I am exceedingly well pleased with the results. Dr. Case, of Jackson, Mich., U. S., about two years ago spoke of nearly the same method as giving very good results, and within the past year in England the same method has found favor to some extent.

The Knapp system of solid gold crown merits notice from an artistic stand-point, and recommends itself to those who have the time and inclination to do what may just as easily be accomplished in one-fourth the time, and that, too, in many cases with less sacrifice of dental tissue.

The porcelain-faced crown, built up by the same process, I tried more than a year before Dr. Knapp's method was known, but I discarded it as a time-waster, and in preference use the method previously spoken of.

I have found that the deep collars or bands, which at first were supposed to be requisite to the success of an operation of this kind, to be quite unnecessary, a narrow one being better and much more compatible with the anatomical surroundings, and promises a greater success than experience has proved deep bands capable of; the reason for this is, there is less irritation produced while fitting them, and much less danger of the rupture of the dental ligament and the subsequent recession of the gum, thereby causing the band to be severely in evidence, and through its exposure, combined with gingival irritation, a nidus is afforded for deleterious products that become factors in defeating the objects of the operation.

I think in some cases the band may be entirely dispensed with to advantage; where the root is an indifferent one, it may readily be restored with amalgam and mounted with a crown; this in many cases will restore to usefulness a root that may long have been out of service. I have resorted to this method with advantage, restoring quite satisfactorily an otherwise faulty articulation and obviating the necessity of a plate.

The Bonwill crown offers in many cases facilities afforded by but few others, combining appearances, range of adaptability, and comparative ease of adjustment that goes far towards making it a desirable acquisition. In connection with crown-work, the Gates-Glidden drill has proved in no small manner to be a factor in our successes. I believe in a thorough preparation of the root, and the fitting of a suitable pin or pins to each case. I do not use a stereotyped pin, but frequently have to make one to suit a particular case, for by having a pin adapted to the size and contour of a root contributes in no small measure to the ultimate results of an operation. While it is well to be conversant with the various kinds of crowns, I think in practice we will necessarily confine ourselves to a very few, for it is much better to become experts in a few methods than to dabble in many, as the end desired is the same in all cases, and in proportion to the complexity of the operation, in proportion is it removed from the average operator. Simplicity of process, when producing satisfactory results, will be admitted on all hands the desideratum of the active practitioner. In our public utterances we are more likely to speak of our successes than we are to descant upon our failures, though who of us here assembled have not made them? And having made them, I hope we have turned them to account by noting and analyzing for the cause that produced the undesired results, and having done this, let us so shape our future work by formulating a definite line of practice whereby we shall reduce to a minimum the element of failure, or modified success that must ever be present where indecision or absence of a definite plan accompany the earlier stages of an operation.

The famous aphorism, "be sure you are right, then go ahead," only needs a slight modification to be quite applicable to our profession,—be sure in your diagnosis, then proceed with the treatment. I cannot help but think the perfection or elaboration to which the mechanical part of bridge-work has arrived has to quite an extent been at the expense of its utility, and by mentally deficient enthusiasts its greatest benefits have thereby been debased.

An utter ignoring of the physiological principles and processes that form the substratum of all dental operations seems to me to be more the rule than the exception in this class of operations, and by the—in many cases—tendency towards mechanical novelty. Physical requirements have been forced into the background in a manner that does not redound to the credit of the members of a profession whose foundations are supposed to be built upon the bed-rocks of anatomy, histology, physiology, and chemistry. This belief is more thoroughly enforced upon me when I see in our journals cuts and details of wonderful appliances supported upon foundations of the flimsiest description, showing their advocates to be entirely destitute of a knowledge of the elementary principles of mechanics, which inferentially, if not conclusively, bespeaks a poverty of general fundamental detail that in almost any calling should debar their utterances from gaining publicity, to the detriment and misleading of the younger members of our profession.

There are very few superfluities in nature; therefore we must conclude originally our dental complement was perfect for the purpose intended; that we can and do succeed fairly well in living with imperfect dentures is no argument in favor of superimposing an entire set of teeth upon three or four faulty roots, and when men get up in societies and state that "bridge-work can be successfully inserted upon a few loose roots that are in an advanced state of pyorrhœa alveolaris, thereby rendering them firm and solid," it is time for the profession to call a halt upon such fallacies, and inquire if some asylum has not discharged one of its late patients as "incurable."

Absurd ideas and statements always have, and probably always will have, advocates; but I hope there is sufficient mental ballast and moral back-bone in the profession to negative and overcome such fictional emanations, and instead of allowing them to pass current by kindly ignoring them, denounce them in an unmistakable manner, thereby locating a rock and preventing the stranding of some professional brother who may be unwittingly drifting within its treacherous influence.

The cases that present themselves for our consideration are of such a varied nature that special description would avail but little here, each case necessarily having to stand upon its own merits; but with a clear conception of the anatomical, physiological, functional and mechanical aspects of the case, and a moral motive force impelling towards the greatest good to our patients, there can be but one outcome to our efforts.

I can only speak positively of fixed bridge-work, not having had any personal experience with the movable kind. The reason I have not made movable work is owing to the few cases where it is possible to secure anchorages or abutments upon parallel lines; where these are not naturally so, it is an operation involving a great deal of time and patience for their artificial production, this, combined with the disadvantages incidental to the enforced use of soft gold or platina in the construction of the work that must necessarily have a certain amount of play to insure of its removal, is in my mind an argument against its general adoption. There are also other points upon which its advisability may be questioned that will present themselves upon a little study of the subject.

Fixed bridge-work has advantages over plate-work that cannot be denied, but, as I have before stated, it is a full appreciation of the requirements of the case that conduces to its success; it can be so constructed as to be as cleanly and as comfortable as the natural teeth; and if, as is often necessary, the articulation is modified to suit the indivividual case, a source of comfort and utility is afforded the patient impossible by any other means.

The various means for the retention of crowns and bridge-work are commensurate with the work itself. Amalgam, gutta-percha and cement may all be used to advantage; an ethereal solution of sulphur may also be used. I prefer for both crowns and bridge-work a fine-grained, slow-setting phosphatic cement.

Never use oxychloride of zinc for setting crowns, the chemical action is so rapid and pronounced that the destruction of the adjacent soft tissues is sure to follow, accompanied with much pain.

In conclusion, if your work is based upon correct principles, the work mentioned in this paper will prove a remunerative as well as a successful and interesting portion of your practice.—*International Dental Journal.*

